# Low-dose erythromycin in pediatrics: Formulation and stability of 20 mg hard gelatin capsules

**DOI:** 10.1371/journal.pone.0282164

**Published:** 2023-02-24

**Authors:** Patrick Thevin, Christophe Curti, Alexandre Benech, Christophe Jean, Edouard Lamy, Caroline Castera Ducros, Nicolas Primas, Pierre Bertault-Peres, Patrice Vanelle

**Affiliations:** 1 Service central de la qualité et de l’information pharmaceutiques (SCQIP), Pharmacy Department, AP-HM, Marseille, France; 2 CNRS, Institut de Chimie Radicalaire ICR, UMR 7273, Equipe de Pharmaco-Chimie Radicalaire, Aix Marseille Univ, Marseille, France; 3 Pharmacie Sainte Marguerite, Pharmacy Department, AP-HM, Marseille, France; 4 UMR 7287 CNRS, Institut des Sciences du Mouvement ISM, Faculté des Sciences du Sport Marseille, Aix Marseille Univ, Marseille, France; Cairo University, EGYPT

## Abstract

**Objective:**

Erythromycin is a macrolide antibiotic that is also prescribed off-label in premature neonates as a prokinetic agent. There is no oral formulation with dosage and/or excipients adapted for these high-risk patients.

**Methods:**

Clinical studies of erythromycin as a prokinetic agent were reviewed. Capsules of 20 milligrams of erythromycin were compounded with microcrystalline cellulose. Erythromycin capsules were analyzed using the chromatographic method described in the United States Pharmacopoeia which was found to be stability-indicating. The stability of 20 mg erythromycin capsules stored protected from light at room temperature was studied for one year.

**Results:**

20 mg erythromycin capsules have a beyond use date not lower than one year.

**Conclusion:**

20 milligrams erythromycin capsules can be compounded in batches of 300 unities in hospital pharmacy with a beyond-use-date of one year at ambient temperature protected from light.

## Introduction

Erythromycin is a macrolide antibiotic with a bacteriostatic effect *in vivo* [1]. Immunomodulating and anti-inflammatory effects have also been described in several studies and has been correlated with inhibition of the synthesis and the release of pro-inflammatory cytokines [2–4].

Like many antibiotics, macrolides can cause disruption of the gut flora, leading to adverse digestive effects such as diarrhea. Among macrolides, erythromycin has the higher frequency of digestive adverse effects. 15% to 20% of patients may experience these adverse events [5].

Erythromycin has also been described as a prokinetic agent, capable of increasing gastro-intestinal motility through an agonist effect on motilin receptors [6]. This pharmacological property leads to stimulation of the third phase of the migrating motor complex and may also results in an orexigen effect [7].

Premature neonates, especially those with a very low birth weight (less than 1,000 g), exhibit gastro-intestinal hypomotility responsible for food intolerance. Such immaturity of gastro-intestinal function is clinically defined by an inability to digest enteral alimentation, by an increase of gastric residues and an abdominal distention with or without vomiting [8]. These symptoms may affect growth and weight gain and may be complicated by a necrotizing enterocolitis. Food intolerance may also increase the duration of parenteral nutrition and the occurrence of parenteral nutrition associated cholestasis [9].

Therefore, erythromycin administration as a prokinetic agent has been evaluated in several studies in pediatric practice (Table 1).

**Table 1:** Erythromycin clinical studies in pediatrics as a prokinetic agent.

Entry	Dosage	Duration (days)	Route	Results^a^	P(atients^b^
1 [10]	15 mg.kg^-1^ t.i.d.	7	IV	NE	76 (35 / 41)
2 [11]	12 mg.kg^-1^ q.i.d.	14	*Per os*	NE	73 (36 / 37)
3 [12]	2.5 mg.kg^-1^ q.i.d.	10	*Per os*	E	43 (22 / 21)
4 [13]	1.5 mg.kg^-1^ q.i.d.	8	*Per os*	NE	27 (15 / 12)
5 [14]	5 mg.kg^-1^ t.i.d.	7	*Per os*	NE	24 (13 / 11)
6 [15]	1 mg.kg^-1^ t.i.d.	unknown	*Per os*	E	60 (30 / 30)
7 [16]	10 mg.kg^-1^ q.i.d. for 2 days, then 4 mg.kg^-1^ q.i.d.	7	*Per os*	E	46 (23 / 23)
8 [17]	12.5 mg.kg^-1^ q.i.d.	14	*Per os*	E	56 (27 / 29)
9 [18]	12.5 mg.kg^-1^ q.i.d.	10	*Per os*	E	57 (29 / 28)
10 [19]	12.5 mg.kg^-1^ q.i.d.	14	*Per os*	E	182 (91 / 91)

^a^ NE = not effective, E = effective

^b^ Total number of patients (treated / placebo)

More than 10 years ago, a meta-analysis was unable to prove the efficacy of erythromycin for the prevention and treatment of food intolerance, [20] but concluded that further studies were needed. The most recent and large study [19] that concluded on the efficacy of oral erythromycin was not included in this meta-analysis (probably because it was published shortly before the meta-analysis). This study also concluded that oral administration should be preferred to parenteral administration for tolerance purpose. The authors considered erythromycin pharmacokinetic to choose dosage regimen [19].

According to these results, low-dose erythromycin is used in our hospital as a treatment of premature neonates’ gastro-intestinal hypomotility. There is no oral formulation with dosage and/or excipients adapted to these high-risk patients. Although low-dosage suspensions may be marketed in some countries, they are not suitable for neonates’ administration (high osmolality, parabens used as preservatives…).

*In vivo*, oral erythromycin therapy suffers from several limitations: erythromycin is very unstable in acidic medium, has a very low solubility, a short half-life and low bioavailability. To overcome these inconveniences, a wide variety of complex erythromycin formulations (liposomes, niosomes, micelles…) have been studied, but none is yet commercialized [21]. In current pediatric practice, hospital pharmacists’ choices are often limited to liquid oral form (suspension) or dry oral form (capsules).

Our pharmaceutical department has developed 20 mg erythromycin hard gelatin capsules suitable for this pediatric indication, which can be opened and poured into 2 mL of a liquid before administration. Microcrystalline cellulose has been used as a diluent, as an excipient that is not absorbed systemically (non-toxic) [22]. Moreover, microcrystalline cellulose is not soluble and therefore has no influence on the resulting osmolality when the capsule is dispersed in a liquid.

First, we will present herein and discuss the Randomized Controlled Trials that have studied similar indications for low-dose erythromycin. Then, we will present our compounding formula and its stability study. Our study shows that 20 mg erythromycin capsules can be stored for at least one year protected from light at room temperature.

## Material and methods

### Erythromycin capsule compounding

Three experimental batches of 50 capsules containing 20 mg of erythromycin were formulated with using a weight-based method. Traditionally, our capsules were compounded with a volume-based method, but recent findings have encouraged us to use a weight-based method for hospital preparations [23]. First, the tapped density of the erythromycin/cellulose mixture was found to be equal to 0.460 g.mL^-1^. One gram of pharmaceutical-grade erythromycin (Cooper^®^ France) and 3.8 grams of microcrystalline cellulose (Cooper^®^ France) were weighed on a qualified precision balance (Precisa XT220A, Precisa^®^). Approximatively 2 mg ± 0.4 mg (one spatula tip) of red carmine (Fagron^®^ France) were added as a homogenization tracer. The mixture was transferred to a mortar for gentle mixing with a pestle. Fifty hard gelatin capsules (size 4, ivory) were placed on a manual capsule-filling machine (ProFiller 3700, LGA^®^). The caps were separated from the empty bodies and the entire mixture was inserted into the capsule bodies. The caps were then replaced, and the capsules were sealed. Routinely, these quantities were multiplied by 6 to obtain batches of 300 hard gelatin capsules.

### HPLC method validation and analysis

Erythromycin capsules were analyzed using the chromatographic method described in the United States Pharmacopoeia [24] and validated to be stability-indicating in our laboratory. The chromatographic method used an automatic High Performance Liquid Chromatography with UV Diode Array Detector (HPLC-UV-DAD) system with a PLRP-S, 1000 Å, 8 μm, 4.6 x 250 mm column (Agilent^®^). The mobile phase consisted of a mixture of phosphate buffer adjusted to pH 9 (3%, v/v), *tert*-butyl alcohol (10%, v/v), acetonitrile (27%, v/v) and ultrapure water (60%, v/v). It was filtered through a Millipore 0.45 μm cellulose filter and used in isocratic mode with a flow of 2 mL.min^-1^ for 30 min. The wavelength for erythromycin detection was 215 nm, the injection volumes were 20 μL and the column temperature was 60°C. Linearity was determined with eight concentrations ranging from 0.25 to 8.00 mg.mL^-1^ prepared in sextuplicate from erythromycin secondary standard dissolved in methanol. Repeatability was determined from within-day variation measurements, whereas intermediate precision and accuracy were determined from between-day variation measurements.

Erythromycin secondary standard (PHR1039-1G, Sigma Aldrich^®^) was used after comparison with erythromycin United States Pharmacopoeia primary standard (1242000, Sigma Aldrich^®^).

The forced degradation studies were conducted on an 8.00 mg.mL^-1^ erythromycin solution in methanol diluted with ultrapure water or reactant to reach a final theoretical concentration of 4.00 mg.mL^-1^. The studied experimental conditions for erythromycin degradation in aqueous solution were acidity (HCl 0.1 M final concentration 0.05 M), basicity (NaOH 0.1 M final concentration 0.05 M), heat (40°C in sealed tube), oxidation (H_2_O_2_ 1% final concentration 0.5%) and light (sunlamp) [25, 26].

After method validation, System Suitability Test (SST) was performed routinely with a 4 mg.mL^-1^ erythromycin secondary standard solution in methanol:phosphate buffer pH7 (25:75) analyzed six times. Conformity criteria were established with the erythromycin monograph if available, or from values found during method validation.

To determine the erythromycin content, a capsule was opened and solubilized with 5 mL of methanol. The solution was placed in an ultrasonic bath for 5 min, vortexed for 5 min and centrifuged at 3000 RPM for 5 min. Two mL of the solution were filtered. The first mL was poured off, and the remaining solution was analyzed by HPLC.

### Disintegration tests

Disintegration tests were performed with an Agilent^®^ 100 automated disintegration apparatus according to the procedure reported both by the European Pharmacopoeia [27] and the United States Pharmacopoeia [28]. Briefly, one capsule was placed in each of six tubes in a 2-basket rack-assembly and the apparatus was operated with water maintained at 37 ± 2°C as immersion fluid. After 30 min, capsules were observed to determine if they were completely disintegrated.

### Stability study

Erythromycin capsules were protected from light and stored in a climatic chamber (25°C, 60% RH). Erythromycin content and degradation products were evaluated at time zero, one week, one month, 2 months, 3 months, 5 months, 8 months and one year after compounding. At each sampling points, 9 capsules from 3 independent batches were analyzed. Disintegration tests were performed on 6 capsules at time zero, one month, 3 months, 8 months, and 12 months. Significant changes in erythromycin content were defined as a 10% change in dosage from its initial value as previously reported for hospital-compounded preparations [29–33].

## Results

### HPLC method validation and analysis

**Fig 1:**
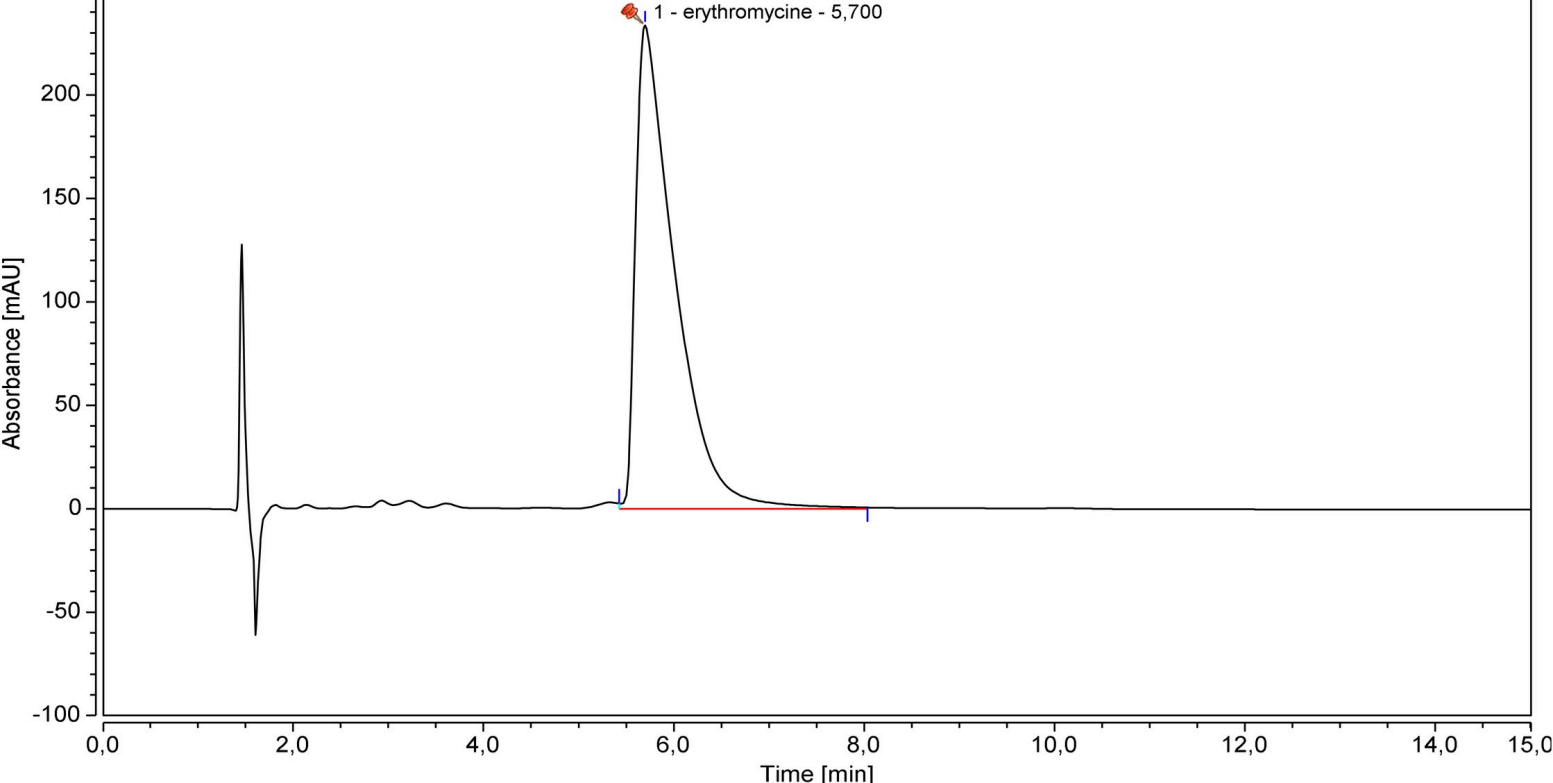
Representative erythromycin chromatogram.

Under the tested experimental conditions, the two lowest concentrations (0.25 and 0.50 mg.mL^-1^) were not found to be linear, so linearity was established between 1.00 and 8.00 mg.mL^-1^. Repeatability, intermediate precision and accuracy results and an example of erythromycin chromatogram are reported in Table 2 and Fig 1.

**Table 2:** Repeatability, intermediate precision and accuracy.

Samples	Repeatability (% RSD within-day) (n = 15)	Intermediate precision (% RSD between-day) (n = 18)	Accuracy (Bias in %) (n = 18)
Erythromycin 3 mg.mL^-1^	0.712	2.796	-2.022
Erythromycin 4 mg.mL^-1^	0.421	1.954	-2.669
Erythromycin 5 mg.mL^-1^	0.414	2.066	-3.063

No interference with the assay method was found during the forced degradation studies. Three degradation products were identified (peaks with relative retention times related to erythromycin: 0.3, 1.4 and 1.6). The results are shown in Table 3 and the chromatograms are also shown in the supplementary material.

**Table 3:** Erythromycin forced degradation study.

Experimental conditions	API degradation (%)	Degradation products relative retention times
Heat (40°C, 60 h)	10	1.4, 1.6
Light (Sunlamp, 132 h)	6	1.6
Oxidation (H_2_O_2_ 0.5%, 50 h)	26	0.3
Acidic (HCl 0.05 M, 60 h)	26	1.4
Alkaline (NaOH 0.05 M, 60 h)	18	1.6

During the method validation, conformity criteria for the SST were established and reported in Table 4.

**Table 4:** System Suitability Test for erythromycin dosing method.

Parameter	Conformity
K’ (capacity factor)	K’ > 2
Rs (resolution)	Rs > 0,8 (USP, erythromycin API)
N (number of theoretical plates)	N > 1000
T (Tailing Factor)	T ≤ 2
RSD of 6 analyses	RSD ≤ 2,0% (USP, erythromycin API)

### Stability study

**Fig 2:**
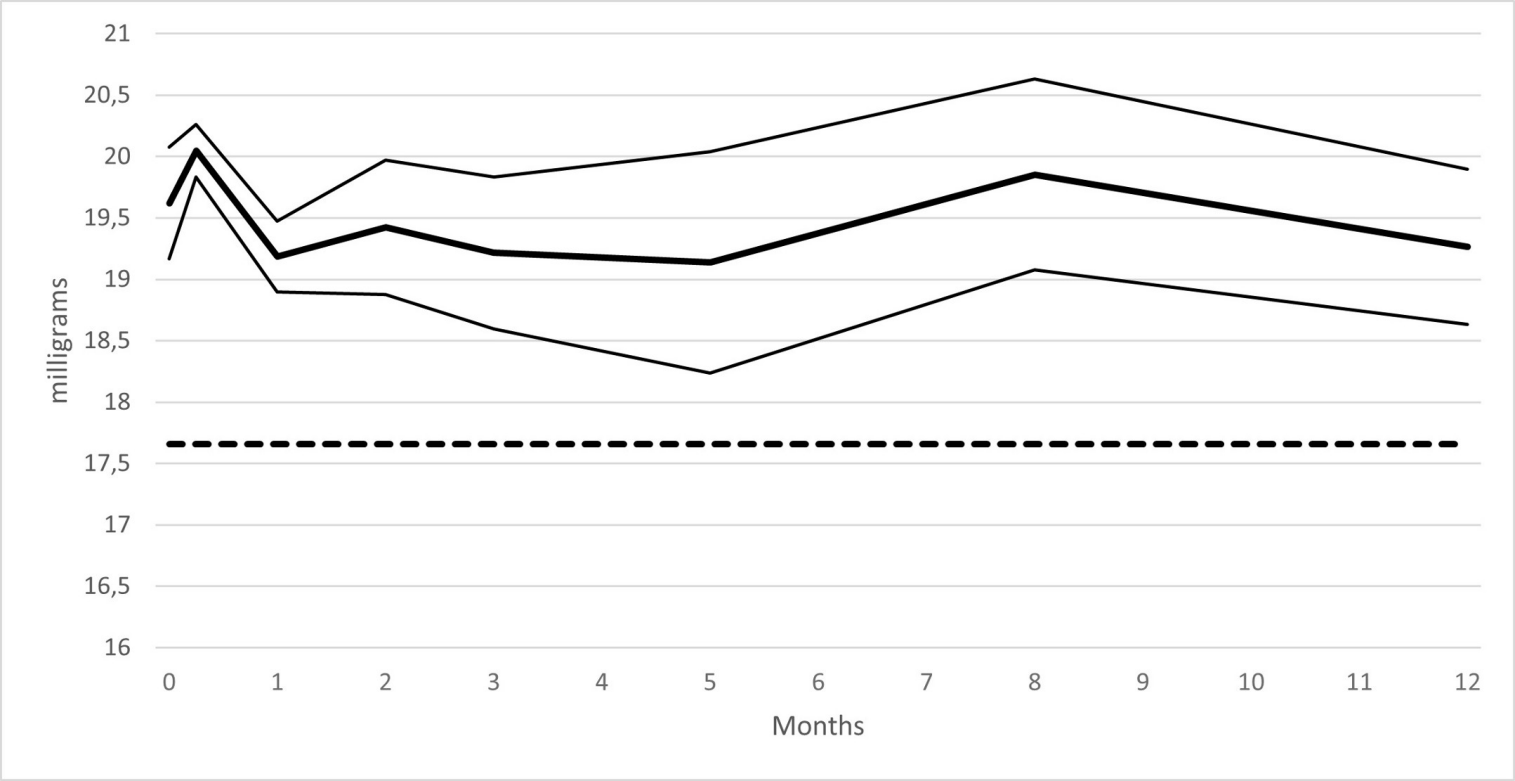
Erythromycin capsules stability study under ambient storage conditions.

Disintegration tests performed on 20 mg erythromycin capsules were in accordance with European Pharmacopoeia recommendations from baseline to 12 months after storage under ambient conditions. The erythromycin content remained above 90% of the initial erythromycin content for 12 months, as shown in Fig 2 (bold line = mean erythromycin content, thick lines = 95% confidence interval). In addition, we did not identify an increase in degradation products during this study.

## Discussion

Erythromycin dosing method described in the United States Pharmacopoeia was validated and found to be stability-indicating.

To our knowledge, the stability of erythromycin compounded formulation has rarely been studied in the literature. Erythromycin delayed-release tablets have been described as stable for 10 days when stored at 40°C, [34] and erythromycin topical formulations have been stable for one month when stored at 25°C [35]. However, in these studies, the quantification methods are not fully described to be stability-indicating as recommend by The International Council for Harmonisation (ICH).

Erythromycin stability has been described as being influenced by pH (acid) and by the presence of water [36]. Therefore, a dry oral form seemed to be of interest for erythromycin. The compounding of batches of 300 hard gelatin capsules was implemented and its feasibility was confirmed by a stability study. 20 mg erythromycin capsules have a Beyond-Use-Date of one year when stored at ambient temperature and protected from light. Since 2020, 43 batches of 300 capsules of erythromycin 20 mg were compounded, controlled (mean erythromycin content, uniformity of dosage units) and dispensed to patients without any report of adverse reactions in our hospital.

Our study suffers from several limitations. First, the main limitation of our method is the low value of found theoretical plates during the analysis, despite column heating at 60°C. During the method validation, we tried higher temperatures, or heating the HPLC tubing, without improvement. Moreover, the column described in the Pharmacopoeia (styrene–divinylbenzene copolymer, 5 to 10 μm in diameter, 4.6 mm X 25 cm, 1000 Å) is very specific, and there are not as many commercial references as classical C18 columns. It was therefore difficult to try other columns in a view to increase the theoretical plates. As the other parameters were correct, we decided to implement the SST with a lower number of theoretical plates than those suggested by the FDA for pharmaceutical analyses.

A second limitation was identified during forced degradation studies. As erythromycin is not soluble in water, methanol was used to make the first stock solution. Therefore, under basic and oxidative conditions, methanol can yield to respectively methanolate and formaldehyde. These products can react with erythromycin and degradation products which will not be observed in the real life could have been obtained through this way.

Despite these limitations, our pediatric erythromycin capsule formulation has many advantages. First, it is adapted to pediatric practice: it can be poured into a low volume of water before administration, and it contains no preservatives or other additives. Then, the unique dosage (20 mg) compatible with a pediatric dosage regimen decreases the risk of dosage error before administration. Finally, capsules can be easily compounded, with raw materials and consumables available worldwide.

## Conclusion

There is an urgent need for age-appropriate medicines for children and especially neonates. A wide variety of promising new and innovative formulations are described, but few are currently marketed. In clinical practices, hospital pharmacists are often forced to choose between a liquid oral medication (solution and suspension) or a dry oral medication (capsule opened and poured into a liquid). For pediatric indications, we recommend the compounding of 20 mg erythromycin capsules.

Batches of 20 mg erythromycin capsules can be stored for at least one year protected from light at room temperature. This long-term stability allows hospital pharmacists to compound and store erythromycin capsules and to avoid emergency preparation (overnight or during weekend). In addition, as erythromycin stability in liquid media remains unknown and can be assumed to be low, capsules opened and poured into a liquid should be preferred to oral liquid medications.

## Supporting information

S1 File Forced degradation study. (DOCX)
