# Supplementary material for: Low-dose erythromycin in pediatrics: Formulation and stability of 20 mg hard gelatin capsules
Source: PLoS One. 2023 Feb 24;18(2):e0282164. doi: 10.1371/journal.pone.0282164 (PMC9955640; doi:10.1371/journal.pone.0282164)
Supplement: S1 File — (DOCX) [file pone.0282164.s001.docx]

**Supporting Information**

**Forced degradation study**

**Figure S1. Forced degradation of erythromycin: Heat (40 ^o^C, 60 h)**

**Figure S2. Forced degradation of erythromycin: Light (Sunlamp, 132 h)**

**Figure S3. Forced degradation of erythromycin: Oxidation (H_2_O_2_ 0.5%, 50 h)**

**Figure S4. Forced degradation of erythromycin: Acidic (HCl 0.05 M, 60 h)**

**Figure S5. Forced degradation of erythromycin: Alkaline (NaOH 0.05 M, 60 h)**
